# Multivariate Analysis of Difference Raman Spectra of the Irradiated Nucleus and Cytoplasm Region of SH-SY5Y Human Neuroblastoma Cells

**DOI:** 10.3390/s19183971

**Published:** 2019-09-14

**Authors:** Ines Delfino, Valerio Ricciardi, Lorenzo Manti, Maria Lasalvia, Maria Lepore

**Affiliations:** 1 Dipartimento di Scienze Ecologiche e Biologiche, Università della Tuscia, 01100 Viterbo, Italy; 2 Dipartimento di Medicina Sperimentale, Università della Campania “L. Vanvitelli”, 80100 Napoli, Italy; valerio.ricciardi@unicampania.it (V.R.); maria.lepore@unicampania.it (M.L.); 3 Istituto Nazionale di Fisica Nucleare, sezione di Napoli, 80126 Napoli, Italy; manti@na.infn.it; 4 Dipartimento di Fisica, Università “Federico II,” 80126 Napoli, Italy; 5 Dipartimento di Medicina Clinica e Sperimentale, Università di Foggia, 71100 Foggia, Italy; maria.lasalvia@unifg.it; 6 Istituto Nazionale di Fisica Nucleare, sezione di Bari, 70125 Bari, Italy

**Keywords:** Raman micro-spectroscopy, single SH-SY5Y human cancer cells, Principal Component Analysis, effects of X-rays on cell nucleus and cytoplasm

## Abstract

Previous works showed that spatially resolved Raman spectra of cytoplasm and nucleus region of single cells exposed to X-rays evidence different features. The present work aims to introduce a new approach to profit from these differences to deeper investigate X-ray irradiation effects on single SH-SY5Y human neuroblastoma cells. For this aim, Raman micro-spectroscopy was performed in vitro on single cells after irradiation by graded X-ray doses (2, 4, 6, 8 Gy). Spectra from nucleus and cytoplasm regions were selectively acquired. The examination by interval Principal Component Analysis (i-PCA) of the difference spectra obtained by subtracting each cytoplasm-related spectrum from the corresponding one detected at the nucleus enabled us to reveal the subtle modifications of Raman features specific of different spatial cell regions. They were discussed in terms of effects induced by X-ray irradiation on DNA/RNA, lipids, and proteins. The proposed approach enabled us to evidence some features not outlined in previous investigations.

## 1. Introduction

The potentialities of Raman Spectroscopy (RS) as a diagnostic tool for biofluids, cells, and tissues have been widely demonstrated [1,2,3,4,5,6,7]. RS is a vibrational technique that enables to get information on the functional groups present in the sample under investigation, thus providing a molecular fingerprint of the sample itself. This technique attracts much interest also because it allows one to yield this information also in physiological conditions at various spatial scales, down to the single cell level or single cell region, thanks to the use of Raman micro-spectroscopy approach (μ-RS) [8,9,10]. 

Nowadays, significant efforts are devoted towards developing proper data analysis methods allowing to manage, on one hand, the complexity of the Raman spectra of cells and tissues and, on the other hand, the high number of spectra needed to take into account the intrinsic variability of biological samples. In addition, RS suffers from specific concerns in terms of data processing and analysis, the most relevant being the presence of a broad background which can often swamp the sample signal [11,12,13,14]. The recent development of new methods and approaches for sample analysis and data pre-processing and processing has considerably increased the relevancy of the information contained in the data, facilitating the standardization of methods for data analysis [15,16]. Among the other approaches, spectral difference and multivariate data analysis methods, such as Principal Component Analysis (PCA), are commonly used for the examination of large Raman spectroscopy datasets, allowing the discrimination of different samples or regions of a sample, according to differences in their biochemical content, and identification of the spectral features which manifest the highest degree of variability [1,3,10].

The approach combining μ-RS and proper data analysis methods has attracted particular attention for the study of the effects of ionizing radiation on cell lines, which would particularly benefit from having biochemical information on cell components. μ-RS has been employed to study the effects on cells induced by the γ-ray exposition and, above all, by the X-ray radiation at various doses for tumor and non-tumorigenic cell lines (see [17,18,19,20] and references therein). In particular, relevant radiobiological issues, such as the variation in patient radiosensitivity [18,21], the monitoring of patient’s radioresponse during the course of an extended treatment [22,23], and the failure of current models to predict cell survival at single high doses [17,18,21,24] have benefited from the contribution offered by μ-RS. In addition, it has been demonstrated to be useful also for investigating the radioresponse of various subtypes of breast cancers [22]. This technique has been also recently adopted to evaluate the radiobiological sensitivity of normal human breast cells to proton irradiation [25,26].

Since ionizing radiation acts in a selective manner on the various biochemical constituents of the cells (see [27] and references therein), great efforts have been devoted at designing new approaches enabling to use μ-RS to get information on specific sub-cellular regions of single cells exposed to X-ray radiation. In fact, this is a task that is not easily accomplished by standard biological assays which usually require complex sample preparation procedures. On the contrary, it seems to be one of the peculiar possibilities offered by the μ-RS approach, as recently demonstrated [9,20]. In fact, our previous work [20] has already shown that biochemical changes occurring in single neuroblastoma cells as a consequence of X-ray irradiation with graded doses can be observed by using μ-RS and specific information from nucleus and cytoplasm can be obtained.

In this paper, biochemical changes in the cytoplasm and nucleus region of single SH-SY5Y neuroblastoma cells following the exposure to graded doses of X-rays were investigated by μ-RS by using an alternative approach. Paired spectra from the nucleus and cytoplasm region of the same cell were acquired in order to examine the difference spectra obtained by subtracting the cytoplasm-related spectrum from the corresponding one detected at the nucleus. A proper spectra normalization and subtraction procedure along with the use of interval Principal Component Analysis (i-PCA) on carefully selected intervals was used for the identification of the damaging effects induced by graded doses of X-ray radiation (0, 2, 4, 6, 8 Gy) on cells fixed immediately after irradiation. 

The results showed that it is possible to investigate in a selective way the effects of ionizing radiation on different neuroblastoma cell spatial regions by using the proposed approach. An increase of the signal related to the nucleobases, a decrease of DNA and/or RNA backbone contribution, a protein rearrangement with changes in the secondary structure, and an increase in lipid saturation have been observed. These findings suggest that the proposed approach enables to obtain information on the behavior upon X-ray irradiation of specific modes particularly affecting the difference spectra, thus being complementary to the analysis of bare spectra. 

## 2. Materials and Methods

### 2.1. Sample Preparation and Treatment

DMEM medium, fetal bovine serum, penicillin, L-glutamine, polylysine, and formaldehyde were provided by Sigma-Aldrich Co. (St. Louis, MO, USA) and used without any further treatments. 

SH-SY5Y (American Type Culture Collection, Manassas, VA, USA) is a human cell line subcloned from a bone marrow biopsy taken from a four-year-old female with neuroblastoma. These cells are often used as in vitro models of neuronal function and differentiation. SH-SY5Y cells were cultured in vitro in DMEM medium, supplemented with 20% fetal bovine serum, 1% penicillin and 1% L-glutamine. They were grown at 37 °C, 5% CO_2_ on polylysine-coated glass coverslips (22 × 22 × 0.17 mm^3^), since polylysine coating is known to favor the adhesion of cells on the glass substrate. The concentration of cells was ∼1 × 10^3^ cells/cm^2^; at this concentration, cells were not confluent as to leave sufficient intercellular spaces for measuring the background signal.

A STABILIPAN machine (Siemens, Munich, Germany) was used for X-ray irradiation. X-rays (250 kVp) were produced by a Thomson tube (TR 300F) and filtered by 1-mm-thick Cu foil. Cellular samples derived from one single batch, in order to avoid interbatch variation. Cells exposed to various doses of X-rays (2, 4, 6, 8 Gy) at a dose rate of 1.20 Gy/min were investigated together with unexposed cells (0 Gy).

Immediately after X-ray exposure, slides were removed from the growth medium and fixed in 3.7% paraformaldehyde in PBS for 20’ at room temperature then stored at 4 °C. The fixation procedure is important to store the cells before, during, and after the measurement. Various studies are devoted at investigating the fixation effects on the Raman spectra of cells [28,29]. In particular, Meade et al. showed that a fixation procedure with a low concentration of paraformaldehyde (3.7%) provides the best results and this procedure has been used in many recent studies [19,20,25,26,30,31]. 

### 2.2. Raman Micro-Spectroscopy Measurements

Raman spectra were recorded at room temperature by means of a commercial Raman micro-spectrometer (LabRam, Jobin-Yvon Horiba) by using an external Ar+ laser (λ = 514.5 nm) and a CCD 3000 (Jobin Yvonne-SPEX, Edison, New Jersey) with a spectral resolution of about 4 cm^−1^. A detailed description of the apparatus was reported in [19]. Briefly, an Olympus optical microscope with a 100× oil immersion objective (numerical aperture: 1.4) was used to focus the laser beam on the samples (laser power at the sample = 10 mW). The excitation laser beam passed from the immersion objective through the oil drop to the cover glass and was focused on the cell. In order to limit the contribution of the glass substrate to the Raman signal, the excitation laser beam was carefully focused inside the sample. The glass coverslip with the cells was placed on a microscope slide provided with a well filled with PBS solution (for further details, see the scheme reported as Figure 2 in [20]). The small-size laser spot (about 2 μm) on the focal plane allowed us to probe different regions of the cells. SH-SY5Y cells have a size of about 10 μm with a nucleus size around 4 μm. Accumulation times in the 10–20 s range were used. 

The Raman signal was acquired on morphologically intact cells by focusing the laser beam first on the nucleus (visible by using the oil immersion objective) including also some neighboring cytoplasm, then on the cytoplasm of the same cell close to its edge, thus obtaining a paired couple of spectra (one spectrum from nucleus region and the other form the corresponding cytoplasm region). Finally, the background spectrum was recorded from an empty area of the slide close. A total of 300 spectra from cells were acquired, resulting in 20 paired couples for each of the irradiation conditions (i.e., 0, 2, 4, 6, 8 Gy) and 100 (one for each couple) background spectra. 

### 2.3. Data Analysis

#### 2.3.1. Preliminary Analysis

The whole dataset of the spectra detected from cells was preliminarily processed to eliminate the contribution of external factors by subtracting the corresponding background spectrum from the measured one and, subsequently, performing a piecewise baseline correction to eliminate the contributions due to the fluorescence signal [32]. After this step, the spectra were processed via vector normalization procedure in order to have comparable intensities, using Standard Normal Variate method [16,32]. The vector normalized spectra were then analyzed in terms of convoluted Lorentzian shaped vibrational modes, by performing a best-fit procedure in order to determine optimized intensity, position, and line-width of Lorentzian peaks, starting from an initial manually set configuration of the parameters. Peak deconvolution was carried out using the Microcal Origin software (Version 9.0, OriginLab Corporation, Northampton, MA, USA). The fitting performance was evaluated by means of the χ^2^ parameter.

The fully processed dataset was then analyzed by PCA and i-PCA algorithms [19,20,33], by using the covariance matrix, in order to represent the dataset by orthogonal eigenvectors separately accounting for most of the variance in the original data and to consequently better observe the similarities among spectra. PCA was performed on the full spectral window (500 to 3200 cm^−1^). Then the spectral window was divided into 18 intervals (400–597, 601–816, 820–901, 905–977, 981–1111, 1116–1214, 1218–1299, 1303–1438, 1442–1500, 1505–1599, 1603–1670, 1672–1737, 1741–2820, 2824–2905, 2909–3030, 3035–3100, 3104–3160, 3164–3200 cm^−1^) and the PCA was performed on each interval (i-PCA). The extremes of these spectral ranges were defined taking into account the expected positions of the contributes of biomolecules present in the nucleus and in the cytoplasm, according to what was reported in previous studies [10,18,20] and what was observed in the average spectra (see below) in order to avoid splitting of signal due to a peculiar mode over two or more ranges. 

The results were analyzed for selecting the intervals (named selected intervals) featuring the highest ability in separating between nucleus and cytoplasm by using the first two principal components.

The statistical significance of the separation among cytoplasm-related and nucleus-related clusters of spectra was evaluated by using the approach proposed by Goodpaster and Kennedy, involving the use of Hotelling’s T-square test [34] with a significance level of 1%. The vector normalization procedure, PCA and i-PCA along with Hotelling’s T-square test were performed by using MATLAB^®^ software package (Version 7.6, MathWorks Inc., Natick, MA, USA). 

#### 2.3.2. Difference Spectra and interval-Principal Component Analysis (i-PCA) 

Cytoplasm-related vector normalized spectrum of each paired couple was subtracted from the nucleus-related corresponding spectrum. In so doing, the eventual biochemical differences of components in nucleus and cytoplasm will be evidenced. On this difference spectra data set, the i-PCA was performed on the “selected intervals” (see Section 2.3.1) in order to specifically analyze the differences in the effects induced by X-ray irradiation on the nucleus and cytoplasm regions.

## 3. Results and Discussion

### 3.1. Analysis of Nucleus- and Cytoplasm-Related Spectra

**Figure 1:**
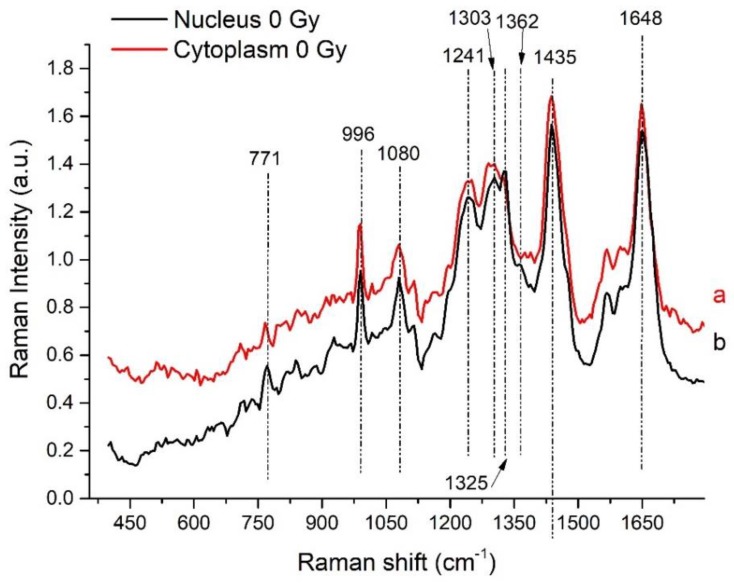
Spectra obtained by averaging all the spectra taken from cytoplasm (red line, a spectrum) and from nucleus (black line, b spectrum) in the fingerprint region (400–1750 cm^−1^) from control cells. Main peaks are labeled; for tentative assignment see Table 1.

**Figure 2:**
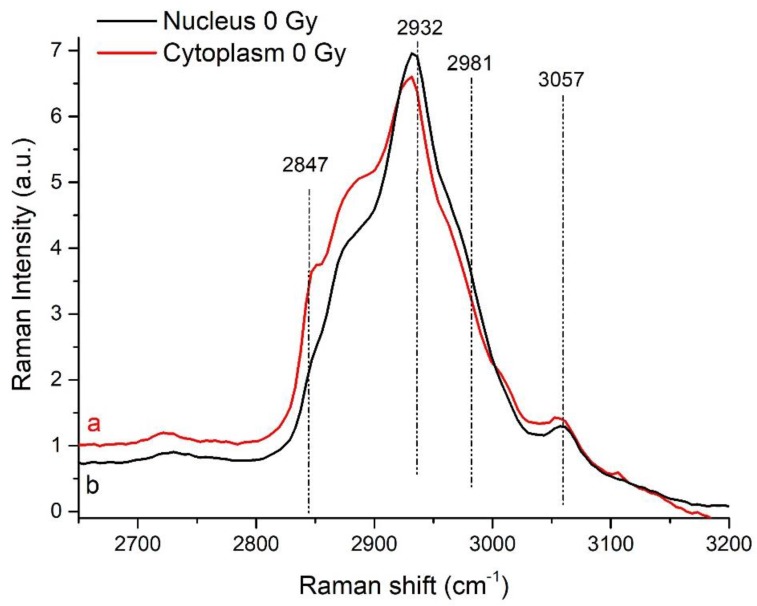
Spectra obtained by averaging all the spectra taken from cytoplasm (red line, a spectrum) and from nucleus (black line, b spectrum) in the high Raman shift region (HSR) (2650–3200 cm^−1^) from control cells. Main peaks are labeled; for tentative assignment see Table 1.

The average vector normalized spectra obtained for both nucleus and cytoplasm regions for control (not irradiated) cells are shown in Figure 1 in the fingerprint region (400–1750 cm^−1^) and in Figure 2 in the high Raman shift region (HSR) (2650–3200 cm^−1^). The main peaks are labelled in the figures. In both the spectral regions, common features can be observed along with differences according to the known different biochemical composition of the two cell regions [9,20]. In the fingerprint region (Figure 1), the most relevant contributions from proteins are present at 1648 cm^−1^ and ≈1240 cm^−1^. The former peak is due to Amide I and is given by the superposition of signals due to various secondary structures of proteins (α-helix, β-sheets and random coil) [19,20] and the latter (the band at ≈ 1240 cm^−1^) is attributed to amide III band. The evident peak at ≈ 1440 cm^−1^ can be thought to be related to CH_2_/CH_3_ bending and scissoring of proteins and lipids as well as CH deformation and also to Adenine/Guanine. As far as concerns other RNA/DNA related contributions, the peaks in the region 1320–1370 cm^−1^ are related to nucleobases with also a contribution from CH deformation. The band at 1090 cm^−1^ is mainly due to PO_2_^−^ symmetric stretching vibrations of the phosphodiester nucleic acid backbone with contributions from CC chains stretching of lipids and CO and/or CC stretching of carbohydrates. The peak at around 770 cm^−1^ can be assigned to Cytosine/Thymine/Uracil ring breathing mode and to O-P-O stretching. 

For the high Raman shift region (2650–3200 cm^−1^), the spectra show the characteristic bands associated to lipids and proteins (Figure 2). In particular, the shoulders detected at around 2980 and 2940 cm^−1^ are assigned, respectively, to the asymmetric and symmetric stretching of the CH_3_ groups arising from cellular proteins and lipids. The bands at around 2890 and 2860 cm^−1^ are assigned to the stretching of the methylene groups of membrane lipids CH_2_, respectively. By using the deconvolution procedure mentioned in the Section 2.3 other specific features were outlined. Similar results are obtained by analyzing the spectra form nucleus and cytoplasm of irradiated cells. The average spectra for all the samples are shown in Figures S1 and S2. The complete scheme of the features observed in the average spectra is reported in Table 1 together with possible peak assignments, in agreement with [17,19,20,35,36,37] and references therein.

The average spectra from nucleus and cytoplasm share the outline features (as detailed discussed in our previous work [20]) showing some differences in their specific characteristics. A higher intensity of the modes in the regions around 1210–1255, 1300–1340 and 2900–2980 cm^−1^ is observed for the nucleus-related spectra than the corresponding ones found in the cytoplasm-related ones. The opposite is the case for modes in the 2840–2900 cm^−1^ region in which the cytoplasm related signal overcomes the one detected from the nucleus. Differences are observed also in the 1610–1670 cm^−1^ region. These observations suggest that the main differences in the average Raman signal between nucleus and cytoplasm are due (i) to secondary structure of proteins (Amide I and Amide III bands), to specific proteins (Tyrosine and Tryptophan) and to protein CH_2_ and CH_3_ stretching modes; (ii) to nucleobases (especially Adenine and Guanine), and (iii) to lipids (CH_2_ and CH_3_ stretching modes).

### 3.2. Multivariate Analysis for Spectral Interval Selection 

In our previous work, the nucleus-related and cytoplasm-related spectra from cells irradiated with various X-ray doses were separately investigated by using PCA and i-PCA. This approach enabled us to outline changes in the position and intensity of some peaks. In particular, as a consequence of irradiation, an increase in the contribution from nearly all the DNA-related features, and α-helix-, phenylalanine-, tyrosine-, CH_3_ asymmetric stretching (in proteins and lipids)-, lipids C_1_-C_2_ stretching-related peaks was discussed along with a decreased contribution from tryptophan-, β-sheet, CH_3_ symmetric stretching (in proteins and lipids)-, CH_3_ deformation (in lipids)-, cholesterol-, and aromatics (in DNA and lipids)-related peaks [20]. 

In the present paper, we introduce a new approach for getting additional information about X-ray irradiation effects on neuroblastoma cells that considers the whole dataset composed by all the paired spectra here discussed as a unique set by taking into account eventual differences in spectra depending on the cell regions and attempting to evidence these differences by analyzing the “difference spectra”, i.e., the spectra obtained by subtracting the cytoplasm-related spectrum to the corresponding nucleus-related one. As a first step, one should understand if there were spectral intervals where the differences between nucleus-related spectra vs. cytoplasm-related ones are higher enough to overcome the observed differences due to irradiation effects. In order to disclose this aspect, we performed the PCA and i-PCA (see Section 2.3.1) on the whole dataset (including all the spectra from nucleus and cytoplasm of control and irradiated cells) considering the two classes of nucleus-related and cytoplasm-related spectra. Then we searched for the spectral intervals where the first two principal components (PCs) enable to distinguish between the two classes. These intervals, if any, would be the ones containing the differences to be further investigated. 

**Figure 3:**
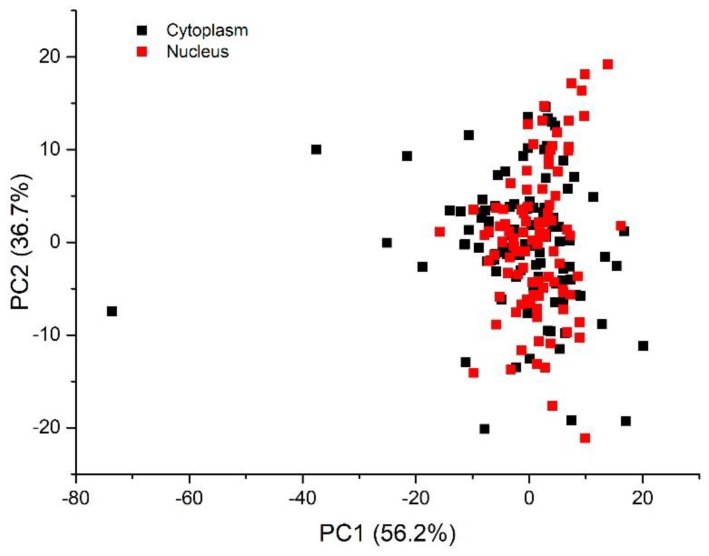
Results of Principal Component Analysis (PCA) on the whole dataset of nucleus- and cytoplasm-related spectra detected for control and irradiated samples. 2D score plot representations (for the two PCs) of the data separately evaluated for cytoplasm-related and nucleus-related spectra.

**Figure 4:**
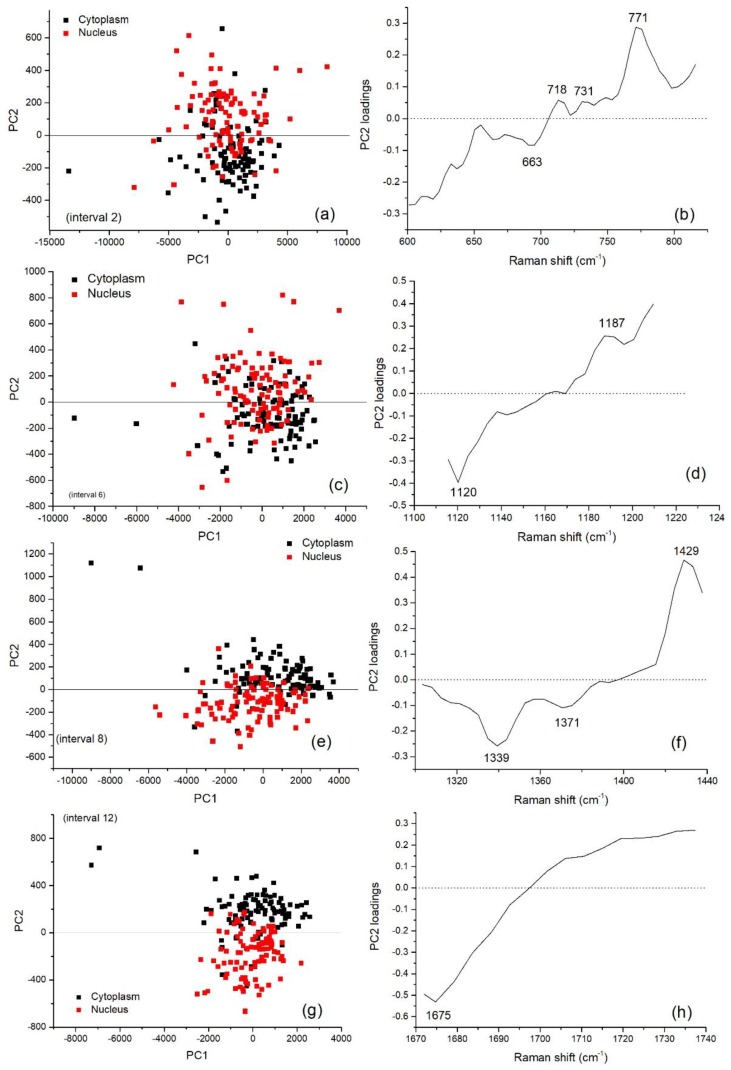
Results of interval Principal Component Analysis (i-PCA) on the whole dataset (control and exposed cells) including nucleus- and cytoplasm-related spectra for the intervals of the fingerprint region in which a separation between spectra from nucleus and cytoplasm region is obtained by using the scores of the first two interval principal components. PC2 vs. PC1 score plot representation of the data for cytoplasm-related (black squares) and nucleus-related (red squares) spectra for the following spectral ranges (intervals): (**a**) 601–816 cm^−1^, (**c**) 116–1214 cm^−1^, (**e**) 1303–1438 cm^−1^, (**g**) 1672–1737 cm^−1^. The corresponding loadings of the second component in the same intervals are reported. PC2 loadings in the following intervals: (**b**) 601–816 cm^−1^, (**d**) 116–1214 cm^−1^, (**f**) 1303–1438 cm^−1^, (**h**) 1674–1737 cm^−1^. According to Hostelling’s T-square test, for all the shown PC score plots, the cluster of nucleus-related scores and cytoplasm-related scores are statistically separated (*p* values lower than 0.0001 were obtained).

**Figure 5:**
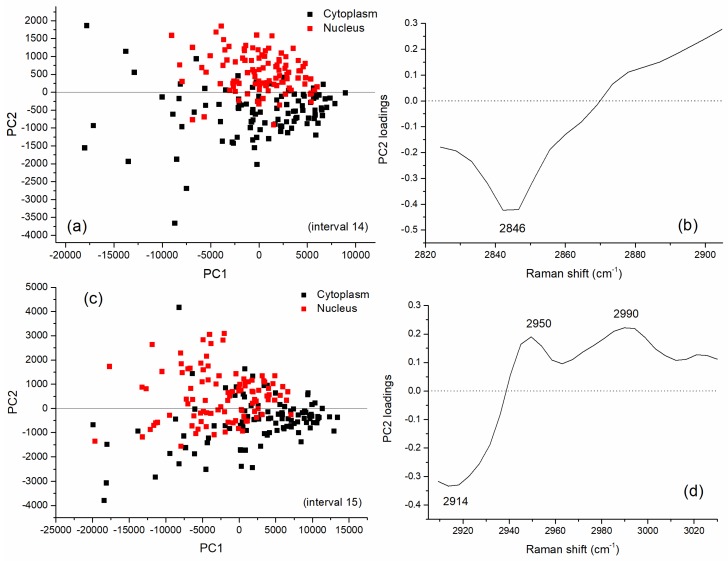
Results of i-PCA on the whole dataset including nucleus- and cytoplasm-related spectra (control and exposed cells) for the intervals of the fingerprint region in which a separation between spectra from the nucleus and cytoplasm region is obtained by using the scores of the first two interval principal components. PC2 vs. PC1 score plot representation of the data for cytoplasm-related (black squares) and nucleus-related (red squares) spectra for the following spectral ranges (intervals): (**a**) 2824–2905 cm^−1^, (**c**) 2909–3030 cm^−1^. The corresponding loadings of the second component in the same intervals are reported. PC2 loadings in the following intervals: (**b**) 2824–2905 cm^−1^, (**d**) 2909–3030 cm^−1^. According to Hotelling’s T-square test, for all the shown PC score plots, the cluster of nucleus-related scores and cytoplasm-related scores are statistically separated (*p* values lower than 0.0001 were obtained).

The results of PCA performed on the full spectral window (500–3200 cm^−1^) are reported in Figure 3 in terms of scores of the first two PCs, accounting for 58% and 24% of the overall variability of the dataset, respectively. By examining the 2D score plot, where the coordinates of each point represent the scores of the considered principal components, it comes out that PCA is not able to clearly separate nucleus and cytoplasm spectra, as confirmed also by performing a Hotelling’s T-square test (*p* > 0.01). On the contrary, when i-PCA is employed for inspecting the complete dataset of nucleus-related and cytoplasm-related spectra of control and exposed cells, in eight spectral ranges (namely: 601–816, 1116–1214, 1303–1438, 1505–1599, 1603–1670, 1672–1737, 2824–2905, 2909–3030 cm^−1^), a clear separation between nucleus- and cytoplasm-related spectra is observed by using the first two PCs, as confirmed by performing the Hotelling’s T-square test (*p* < 0.01). In six of these eight spectral ranges a *p* value lower than 0.0001 was obtained (601–816, 1116–1214, 1303–1438, 1672–1737, 2824–2905, 2909–3030 cm^−1^). The i-PCA analysis performed on these intervals are shown in Figure 4 and Figure 5. In particular, the 2D score plots for the first two components are displayed for the two spectral regions: in Figure 4a,c,e,g the results obtained for the fingerprint region are reported and in Figure 5a,c the results for the HSR region are shown. 

They confirm the ability of i-PCA in extracting details of the spectra in selected intervals and, also, in the present case, in discriminating between spectra from nucleus- and cytoplasm-related spectra. 

In particular, the second principal component is the one being representative of the variation due to the main differences between spectra from the two cell regions, the first one being representative of the intrinsic variability among cells. Accordingly, by inspecting these loadings (reported in Figure 4b,d,f,h and in Figure 5b,d for the fingerprint and the HSR region, respectively), it comes out that the majority of the features characterizing those components enabling to discriminate between nucleus- and cytoplasm-related spectra are mainly assigned to DNA/RNA and proteins. In fact, peaks at 718 and 731 (mainly due to Adenine), 771 (due to DNA/RNA), 1339 (Adenine/Guanine), 1371 (Thymine), 1429 cm^−1^ (Adenine/Guanine and CH_2_ backbone) were outlined to play a relevant role in the discrimination cytoplasm- and nucleus-related spectra along with 1187-cm^−1^ protein-related mode and 1120-cm^−1^ protein and lipids related mode. In addition, features arising from proteins (at 663 and 1675 cm^−1^), lipids/carbohydrates (around 2850 and 2990 cm^−1^) and from proteins and lipids (2914 and 2950 cm^−1^) were observed in the selected components and are thought to be related to significant differences between the two cell-region spectra.

### 3.3. Analysis of Difference Spectra

The analysis reported in the previous paragraph allowed us to evidence that there are spectral intervals where the differences between nucleus-related spectra vs. cytoplasm-related ones are high enough to overcome the differences due to irradiation effects. In addition, these intervals were singled out. Given these results the “difference spectra” can be analyzed by performing i-PCA in the above selected intervals. As a first step, the difference spectra for each paired couple was calculated without introducing any artifact thanks to the employed vector normalization procedure. The difference spectra were then obtained for all the investigated doses and samples.

**Figure 6:**
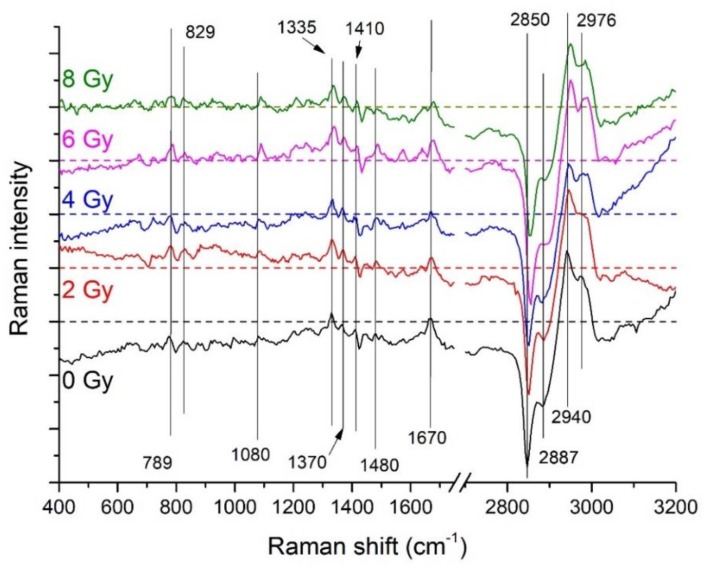
Average difference Raman spectra (for each coupled pair of spectra the different spectrum is obtained by subtracting the cytoplasm-related spectrum to the corresponding nucleus-related one) are reported for the different irradiation doses. Main features are labelled. For the sake of clarity, difference spectra for 2-Gy-, 4-Gy-, 6-Gy-, and 8-Gy-X-ray irradiated cells were shifted along the y axis; for each difference spectra the y = 0 level is represented by the dashed line with the same color of the corresponding spectrum.

The average difference spectra separately obtained for the four investigated irradiation doses and for the control samples (0, 2, 4, 6, and 8 Gy) are shown in Figure 6; the positions of the main observed features being labeled. In the fingerprint region, the majority of the main features (those located at 789, 829, 1089, 1335, 1370, 1410, 1480 cm^−1^) are directly related to DNA/RNA. In addition, significant positive features (intensity in nucleus-related spectrum > corresponding intensity in cytoplasm-related spectrum) are observed in the 1620–1680 cm^−1^ region that is related to amide I and thus to protein secondary structure. In the HSR region, differences between the spectra are also observed in the 2800–3050 cm^−1^ region. The negative difference (intensity in nucleus-related spectrum < corresponding intensity in cytoplasm-related spectrum) observed in the 2800–2900 cm^−1^ region could be due to the different lipid concentration in the two regions. Positive differences around 2930 and 2980 cm^−1^ are also present. Subtle changes in the difference spectra can be observed depending on the irradiation dose that can be better elucidated by performing i-PCA on the selected intervals. 

**Figure 7:**
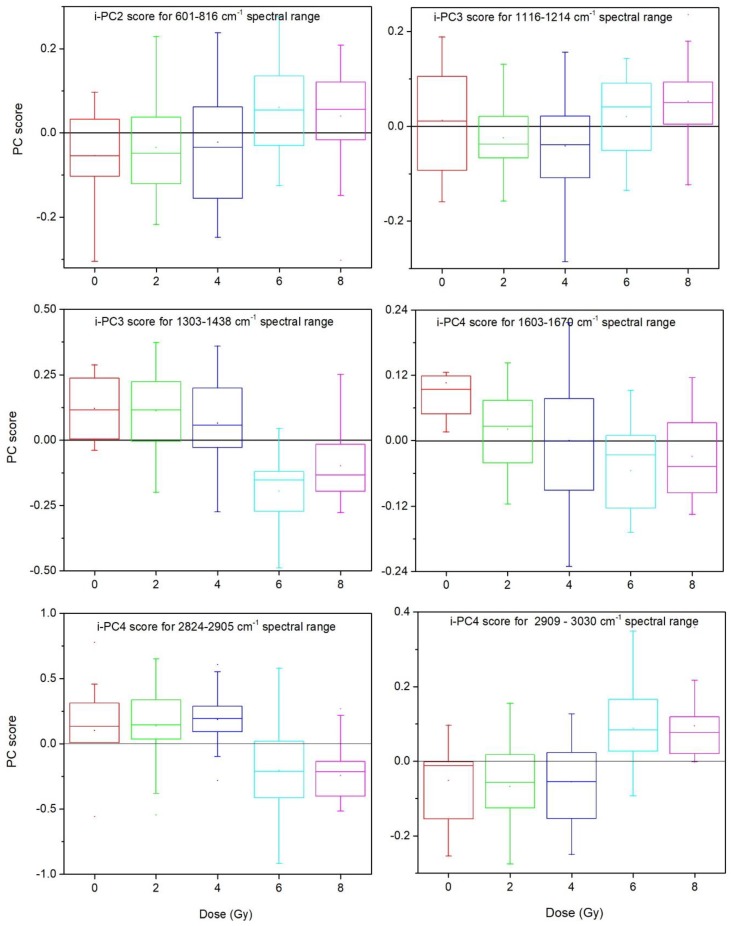
Results of i-PCA analysis of difference spectra for the selected spectral regions: box plot representation of the i-PC scores separately evaluated for each “irradiation treatment”, i.e., 0, 2, 4, 6, and 8 Gy of irradiation dose.

For the outlined intervals the i-PCA results, including-PC scores, loadings, and reconstructed spectra, were examined in order to get details on the dependence of some specific modes on the dose. The intervals showing significant dose dependent changes are the following: 601–816, 1116–1214, 1303–1438, 1603–1670, 2824–2905, 2909–3030 cm^−1^. A summary of the results of this analysis is reported in Table 2 and Figure 7 as obtained by analyzing the PC loadings and scores, and the reconstructed spectra. In particular, the box plot representation of the i-PC scores featuring a dependence on the dose for the selected intervals are shown in Figure 7 for specific components. 

Changes directly depending on the irradiation dose are separately outlined for specific peaks related to proteins, lipids, and carbohydrates as well as features related to DNA and RNA. Regarding DNA/RNA related contributions, an overall increase of the signal related only to the nucleobases (features at 677, 722, 1344, and 1374 cm^−1^) is observed to depend on the irradiation dose. On the contrary, features related to DNA and/or RNA backbone (peaks located at 780, 807, 1325, and 1430 cm^−1^) show decreasing contributions when irradiation dose increases. The PO_2_^−^ related peak at around 780 cm^−1^ decreases as the dose increases. All these evidences can be read as the occurrence of damage to DNA and RNA due to X-ray irradiation leading to a change in the backbone and increased exposition of single nucleobases.

Almost all the protein-related features (767, 1621 cm^−1^) showing changes depending on the dose give decreasing contribution as the X-ray dose increases. The components giving rise to the Amide I band at around 1640 cm^−1^ change in their relative contribution: the features related to the parallel and anti-parallel β-sheet secondary structure (located in the 1625–1640 cm^−1^ region) increases with the dose while the 1661 cm^−1^ peak, related to the α-helix secondary structure, decreases, thus suggesting an increase in the abundance of the β-sheet secondary structure with respect to the α-helix [17]. Regarding protein, lipid, and carbohydrate contributions, an increase in the signal intensity related to the CH_3_-asymmetric stretching (2955 cm^−1^) is observed along with a corresponding decrease in the one due to the CH_2_-stretching (linked to the 2940 cm^−1^ feature, assigned to asymmetric stretching). The contribute of the lipid/carbohydrate-only related mode located at 2860 cm^−1^ (CH_2_-symmetric stretching) also decreases. Some of these results confirm what observed by separately analyzing nucleus-related and cytoplasm-related spectra, as reported in [20]. In particular, by analyzing the difference spectra the features giving similar contributions to both cytoplasm- and nucleus-related spectra are not outlined since they do not significantly influence the overall variation of difference spectra. Accordingly, the here outlined features are related (i) to modes of components giving a high contribution in only one region (such as the outlined DNA/RNA related features and lipid/carbohydrate -related modes) or (ii) to modes having different behavior in the two regions, or (iii) to low-intensity modes not evident in the bare spectra. The features located at 655, 677, 807, 2860, 3003 cm^−1^ are of particular relevance because they have not been outlined in the previous study. 

An overall analysis of the observed changes points out that the analysis of difference spectra further confirms that X-ray irradiation induces changes in neuroblastoma cells, with effects on proteins and lipids and, above all, DNA and RNA. In particular, an increase of the signal related to nucleobases, a decrease in the contribution from DNA and/or RNA backbone, a protein rearrangement with changes in secondary structure, and an increase in lipid saturation can be observed. These outcomes are in agreement with previous findings obtained by directly examining cell Raman spectra obtained from neuroblastoma and other cell lines [17,19,20,38]. For instance, the increase in the β-sheet configuration (that occurs in the proteins at the expenses of the α-helix a consequence of X-ray irradiation) has been already observed in other cellular systems [17]. An increase in lipid saturation can be also hypothesized, in agreement with previous studies [20,39,40].

## 4. Conclusions

The X-ray irradiation effects on the nuclear components of single SH-SY5Y human neuroblastoma cells irradiated by graded X-ray doses (2, 4, 6, 8 Gy) were investigated by Raman micro-spectroscopy. The study was carried out by using a properly designed acquisition and data analysis protocols, including the acquisition of coupled spectra from the nucleus and cytoplasm and the use of PCA of the difference spectra. 

The analysis by i-PCA of the nucleus-cytoplasm difference spectra allowed us to unravel the subtle modifications, due to X-ray irradiation, of Raman features specific of components with different relative abundances in the two regions or to modes with low intensity that are not evident in the bare spectra and that were not examined in our previous investigation [20]. They were discussed in terms of possible damage induced by X-ray irradiation to the single cellular components. In particular, the effects on DNA/RNA backbone and single nucleobases were described along with those occurring on lipids and proteins.

It is worthwhile to note that the experimental set-up and the conditions here employed for the acquisitions are well suited for in vivo investigations, because of the possibility of studying cells immersed in buffer, the low laser power, and the short acquisition time. 

## Figures and Tables

**Table 1:** Raman peaks observed in the spectra from nucleus and cytoplasm of fixed control and irradiated cells, with assignments in accordance with the data reported in the literature. (see [17,19,20,35,36,37] and references therein); abbreviation: sym. = symmetric, asym. = asymmetric, str. = stretching, br. = breathing, tw. = twist, in-p. = in-plane, o-p. = out-of-plane, ben. = bending, def. = deformation, bk. = backbone.

Peak (cm^−1^)	DNA/RNA	Protein	Lipid/Carbohydrate
3057	Aromatics		Aromatics
2994–3008			CH str.
2950–2980		CH_3_ asym. str.	CH_3_ asym str
2918–2940		CH_2_ asym. str.	CH_2_ asym. str.
2885			CH_3_ sym. str.
2860			CH_2_ sym. str.
1640–1670		Amide I (α-helix, β-sheet, coil)	C = C sym. str.
1621		Tyrosine/Tryptophan C = C	
1594–1608		Phenylalanine/Tyrosine C = C	
1572–1581	Adenine, Guanine		
1487	Adenine, Guanine		
1447–1460		CH_2_/CH_3_ ben., CH def.	CH_2_ sc., CH def.
1421–1430	Adenine, Guanine, CH_2_ bk.		
1374	Thymine		
1338–1345	Adenine, Guanine	CH def.	
1326	Guanine	CH def.	
1309	Adenine	Amide III	CH_2_ tw.
1297			CH_2_ tw.
1256–1263		Amide III (α-helix)	CH_2_ def.
1248–1255		Amide III (β-sheets)	
1219–1235		Amide III (random coil)	
1190–1204		Tryptophan/Tyrosine/Phenylalanine CH sym. str., CH ben.	
1168–1178		Phenylalanine/Tyrosine, CH ben.	
1123		CN sym. str.	CC asym. str., CO str.
1090–1098	O-P-O str.		CC chain sym. str., CO, C = C str.
1051–1066	CO sym. str.		CC chain sym. str., CO, C = C str.
1020–1032		Phenylalanine, CH in-p. ben.	
996–1003		Phenylalanine sym. ring br.	
973–986	PO_2_^3-^ sym. str.	CC sym. str., β-sheets	CH ben.
955–963			CH_3_ def.
932–938		CC sym. str., α-helix	COC glycos. bond
883	cDNA		CCN^+^ sym. str., COC ring
870			C_1_ - C_2_ str.
849–853		Tyrosine ring br. Proline CC str.	
823–840	O-P-O bk.	Tyrosine ring br.	
807–811	O-P-O str. RNA		O-P-O str.
770–785	Cytosine/Thymine/Uracil ring br. O-P-O str.		
753–767		Tryptophan ring br.	
722–728	Adenine		Choline
704			Cholesterol
677	Thymine, Guanine		
655		Tyrosine (skeletal)	

**Table 2:** Raman features of difference spectrum dataset showing significant dependence on the dose (see text). Abbreviation: sym. = symmetric, asym. = asymmetric, str. = stretching, br. = breathing, tw. = twist, in-p. = in-plane, o-p. = out-of-plane, ben. = bending, bk. = backbone, d=DNA/RNA, p = proteins, l/c = lipids/carbohydrates.

Peak (cm^−1^)	Assignment	Dependence upon Increasing Dose
3003	**l/c** (CH str.)	Contribution ***decreases***
2955	**p and l/c** (CH_3_ asym. str.)	Contribution increases
2940	**p and l/c** (CH_2_ asym. str.)	Contribution ***decreases***
2860	**l/c** (CH_2_ sym. str.)	Contribution ***decreases***
1640–1670	**p** (Amide I -α-helix, β-sheet, coil) and **l/c** (C = C sym. str.)	1640 cm^−1^ contribution increases; 1661 cm^−1^ contribution ***decreases***
1621	**p** (Tyrosine/Tryptophan C = C)	Contribution ***decreases***
1430	**d** (Adenine, Guanine, CH_2_ bk.)	Contribution ***decreases***
1374	**d** (Thymine)	Contribution increases
1344	**d** (Adenine, Guanine)	Contribution increases
1320–1330	**d** (Adenine, Guanine, CH_2_ bk.)	Contribution ***decreases***
807	**d** (O-P-O str. RNA)	Contribution ***decreases***
780	**d** (Cytosine/Thymine/Uracil ring br., O-P-O str.)	Contribution ***decreases***
767	**p** (Tryptophan ring br.)	Contribution ***decreases***
722	**d** (Adenine) and **l/c** (Choline)	Contribution increases
704	**l/c** (Cholesterol)	Contribution increases
677	**d** (Thymine, Guanine)	Contribution increases
655	**p** (Tyrosine skeletal)	Contribution increases

## Supplementary Materials

The following are available online at https://www.mdpi.com/1424-8220/19/18/3971/s1, Figure S1: Spectra obtained by averaging all the spectra taken from cytoplasm. separately evaluated for each “irradiation treatment”, i.e., 0 Gy, 2 Gy, 4 Gy, 6 Gy and 8 Gy of irradiation dose. For the sake of clarity, spectra are shifted along the y-axis, Figure S2: Spectra obtained by averaging all the spectra taken from nucleus. separately evaluated for each “irradiation treatment”, i.e., 0 Gy, 2 Gy, 4 Gy, 6 Gy and 8 Gy of irradiation dose. For the sake of clarity, spectra are shifted along the y-axis.
