# Supplementary material for: Multivariate Analysis of Difference Raman Spectra of the Irradiated Nucleus and Cytoplasm Region of SH-SY5Y Human Neuroblastoma Cells
Source: Sensors (Basel). 2019 Sep 14;19(18):3971. doi: 10.3390/s19183971 (PMC6766837; doi:10.3390/s19183971)
Supplement: Supplementary file 1 [file sensors-19-03971-s001.pdf]

Supplementary materials

Multivariate Analysis of Difference Raman Spectra of the Irradiated Nucleus and Cytoplasm Region of SH-SY5Y Human Neuroblastoma Cells

Ines Delfino [1,*], Valerio Ricciardi [2,3,#], Lorenzo Manti [3,4], Maria Lasalvia [5,6] and Maria Lepore [2]

[1] Dipartimento di Scienze Ecologiche e Biologiche, Università della Tuscia, 01100 Viterbo, Italy; delfino@unitus.it (I.D.)
[2] Dipartimento di Medicina Sperimentale, Università della Campania "L. Vanvitelli", 80100 Napoli, Italy; valerio.ricciardi@unicampania.it (V.L.), maria.lepore@unicampania.it (M.Le.)
[3] Istituto Nazionale di Fisica Nucleare, sezione di Napoli, 80126 Napoli, Italy; valerio.ricciardi@unicampania.it (V.L.), manti@na.infn.it (L.M.)
[4] Dipartimento di Fisica, Università "Federico II," 80126 Napoli, Italy; manti@na.infn.it (L.M.)
[5] Dipartimento di Medicina Clinica e Sperimentale, Università di Foggia, 71100 Foggia, Italy; maria.lasalvia@unifg.it (M.La.)
[6] Istituto Nazionale di Fisica Nucleare, sezione di Bari, 70125 Bari, Italy; maria.lasalvia@unifg.it (M.La.)
* Correspondence: delfino@unitus.it
\# Present address: Dipartimento di Matematica e Fisica, Università della Campania "L. Vanvitelli", 81100, Caserta, Italy (V.R.)

Received: 26 August 2019; Accepted: 13 September 2019; Published: date

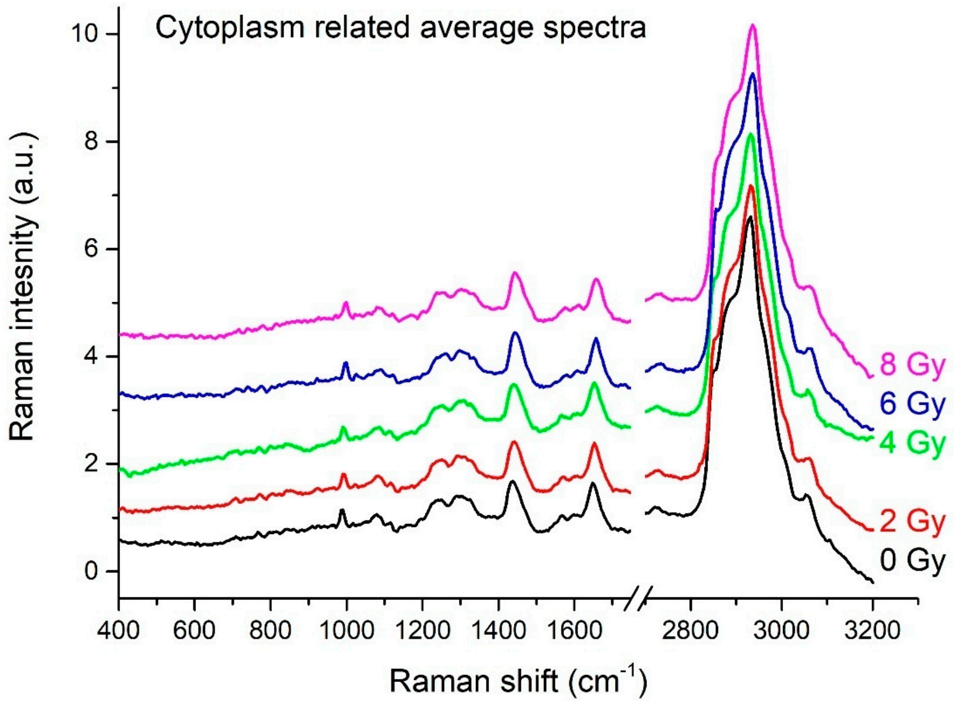

Figure S1. Spectra obtained by averaging all the spectra taken from cytoplasm. separately evaluated for each "irradiation treatment", i.e, 0 Gy, 2 Gy, 4 Gy, 6 Gy and 8 Gy of irradiation dose. For the sake of clarity, spectra are shifted along the y-axis.

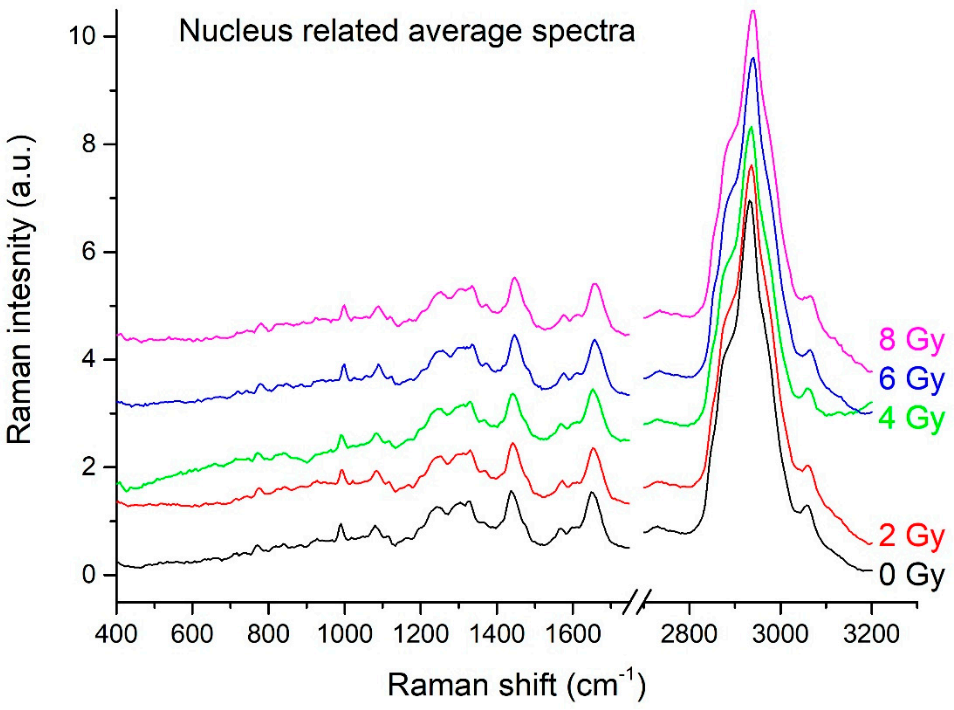

Figure S**2.** Spectra obtained by averaging all the spectra taken from nucleus. separately evaluated for each "irradiation treatment", i.e, 0 Gy, 2 Gy, 4 Gy, 6 Gy and 8 Gy of irradiation dose. For the sake of clarity, spectra are shifted along the y-axis.
